# Selenium Enhances the Growth of Bovine Endometrial Stromal Cells by PI3K/AKT/GSK-3β and Wnt/β-Catenin Pathways

**DOI:** 10.3390/vetsci11120674

**Published:** 2024-12-21

**Authors:** Junsheng Dong, Zi Wang, Fan Fei, Yeqi Jiang, Yongshuai Jiang, Long Guo, Kangjun Liu, Luying Cui, Xia Meng, Jianji Li, Heng Wang

**Affiliations:** 1 College of Veterinary Medicine, Yangzhou University, Yangzhou 225009, China; junsheng@yzu.edu.cn (J.D.); 15051919608@139.com (Z.W.); mx120221011@stu.yzu.edu.cn (F.F.); mz120221610@stu.yzu.edu.cn (Y.J.); yzdxgl@yzu.edu.cn (L.G.); kangjunliu@yzu.edu.cn (K.L.); lycui@yzu.edu.cn (L.C.); mengxia@yzu.edu.cn (X.M.); 2 Jiangsu Co-Innovation Center for Prevention and Control of Important Animal Infectious Diseases and Zoonoses, Yangzhou 225009, China; 3 Joint International Research Laboratory of Agriculture and Agri-Product Safety of the Ministry of Education, Yangzhou 225009, China; 4 International Research Laboratory of Prevention and Control of Important Animal infectious Diseases and Zoonotic Diseases of Jiangsu Higher Education Institutions, Yangzhou University, Yangzhou 225009, China; 5 Guangling College, Yangzhou University, Yangzhou 225009, China; ysjiang0225@yzu.edu.cn; 6 School of Medicine, Yangzhou University, Yangzhou 225009, China

**Keywords:** selenium, bovine endometrial stromal cells, lipopolysaccharide, cortisol, proliferation, apoptosis

## Abstract

The bovine uterus is susceptible to bacterial infections after calving, particularly from *Escherichia coli* (*E. coli*), which often results in endometritis. Additionally, postpartum stress in cows can elevate cortisol levels in the body, inhibiting endometrial regeneration and reducing immune function, thereby further increasing the risk of infection. Selenium (Se) is a common feed additive in dairy farming, known for its anti-inflammatory and antioxidant effects. The aim of this study was to investigate the regulatory role of Se in the growth of bovine endometrial stromal cells (BESCs) under the conditions of LPS-induced inflammatory damage at high cortisol levels. BESCs were treated with 1, 2, 4 μM Se in combination with co-treatment of LPS and cortisol. The results indicated that LPS inhibited the cell viability and reduced the mRNA expression of CTGF, TGF-β1, and TGF-β3. Additionally, LPS increased apoptosis, hindered the cell cycle progression by blocking it in the G0/G1 phase, and suppressed the PI3K/AKT/GSK-3β and Wnt/β-catenin signaling pathways. Furthermore, increased concentrations of cortisol can exacerbate the impacts of LPS on cell proliferation and apoptosis. Conversely, the supplementation of Se promoted cell viability, increased the mRNA expression of TGF-β1 and TGF-β3, and enhanced cell cycle progression, while simultaneously repressing cell apoptosis as well as activating the PI3K/AKT/GSK-3β and Wnt/β-catenin signaling pathways. The above findings demonstrated that Se can promote cell proliferation, reduce cell apoptosis, and aid in the growth of BESCs damaged by LPS under high levels of cortisol. The potential mechanisms may be associated with the regulation of the PI3K/AKT/GSK-3β and Wnt/β-catenin signaling pathways.

## Simple Summary

The cervixes of cows remain open after parturition, increasing the risk of bacterial invasion and thereby predisposing them to endometritis. Meanwhile, bovine endometrial stromal cells are directly infected by bacteria as a result of the destruction of bovine endometrial epithelial cells. During the periparturient period, various stressors can lead to elevated cortisol levels in cows, which can negatively impact cellular growth. Selenium supplementation has the potential to enhance cell proliferation and protect cells from oxidative damage and apoptosis. In this study, we observed that selenium supplementation could enhance cell proliferation and suppress apoptosis damaged by LPS and cortisol. Furthermore, the modulation of cell proliferation and apoptosis by selenium may be accomplished through triggering the signaling pathways involving PI3K/AKT/GSK-3β and Wnt/β-catenin. Thus, supplementing with Se may be an effective strategy for promoting the rapid repair of the endometrium in postpartum dairy cows.

## 1. Introduction

After calving, it is essential for bovine endometrium to undergo uterine involution to ensure that the uterus returns to a normal physiological state and prepares for the next pregnancy [1]. The uteri of nearly all cows are susceptible to contamination by various pathogen microorganisms once the cervix is in an open state [2]. Bacterial infection, particularly by *Escherichia coli* (*E. coli*), is a prevalent cause of endometritis in dairy cows [3,4]. The primary pathogenic factor of *E. coli* is the lipopolysaccharide (LPS) found in its outer membrane. The bovine endometrium consists primarily of epithelial cells and stromal cells, which serve crucial functions in the normal reproductive cycle, implantation, placental formation, pregnancy, and defense against pathogen invasion. Once the bovine endometrial epithelial cells (BEECs) are partly damaged and shed, the direct invasion of pathogenic bacteria and their endotoxins into the bovine endometrial stromal cells (BESCs) is caused [5,6]. Therefore, the rapid growth of the endometrium is imperative for restoring normal physiological function. Compared to BEECs, the production of cytokines and chemokines by BESCs may exert a greater effect due to the proximity of BESCs to the vasculature. Additionally, BESCs outnumber BEECs significantly, making them more susceptible to damage from the endometrial inflammatory response [6].

Cortisol (COR), as a type of glucocorticoid, is a key factor in stress responses, and various stresses can lead to an increase in its levels in the body [7]. During the periparturient period in dairy cows, pregnancy stress, parturition stress, and lactation stress can lead to significant fluctuations in cortisol concentrations [8]. Cortisol plays a crucial role in the function of the corpus luteum during early pregnancy in cows by directly inhibiting the secretion of uterine prostaglandin F_2α_, thereby promoting embryo implantation and early embryonic development [9]. Glucocorticoids exert their inhibitory effects on cell proliferation by inducing cytotoxicity and promoting apoptosis while also leading to cell cycle arrest [10]. High concentrations of cortisol can delay wound healing and suppress immune responses, thereby reducing the endometrial resistance to infection [11]. Selenium (Se) is a vital microelement that is essential for development and various physiological functions, such as immune responses. Geographically, Se deficiency is more prevalent than selenium toxicity [12]. In cases of Se deficiency, both innate and acquired immunity can be compromised [13]. Se deficiency causes thyroid metabolic dysfunction, leading to decreased growth rate and fertility in cattle [14]. Se supplementation can reduce the incidence of metritis and ovarian cysts in periparturient cows, and its administration during the first month of gestation also enhances embryo survival [15,16,17]. Previous studies have shown that Se protein deficiency can increase the expression of inflammatory cytokines in the uterus, mammary glands, and gastrointestinal tract, while also exerting an inhibitory effect on placental proliferation [13,18].

The essential prerequisites for the rapid repair of the endometrium involve a modest inflammatory response, active cell proliferation, reduced cell apoptosis, and accelerated angiogenesis. Numerous reports have confirmed the pivotal importance of the PI3K/AKT signaling pathway in various cellular processes such as proliferation, adhesion, migration, survival, and apoptosis [19,20]. The activated PI3K/AKT signaling pathway can suppress cell apoptosis via upregulating the level of anti-apoptotic protein Bcl-2. Additionally, it triggers a cascade of signaling pathways that enhance cell survival and proliferation. The Wnt/β-catenin signaling pathway is essential for the regeneration of different types of organ tissue. Goad et al. reported on the link between the Wnt/β-catenin signaling pathway and endometrial repair, indicating its activation during the regeneration of endometrial epithelial cells [21]. In the activated state of the Wnt/β-catenin pathway, the translocation of β-catenin into the nucleus of cells modulates the expression levels of downstream target genes, thereby affecting cell proliferation and apoptosis. Notably, genes such as C-Myc and Cyclin-D1 are among those modulated by this pathway [22]. Piras et al. confirmed through proteomic analysis that a large number of differentially expressed proteins are intricately linked to apoptosis in the inflammatory response process of bovine endometrial cells triggered by LPS [23].

The repair process of the endometrium in the cow’s uterus is essential for restoring normal physiological function. Previous research has confirmed that Se can promote the growth of BEECs [24]. Therefore, the aim of this study is to investigate the impact of Se on the enhancement of proliferation and inhibition of apoptosis in BESCs, while elucidating the underlying mechanisms.

## 2. Materials and Methods

### 2.1. Reagents

DMEM-F12 medium (D8900), type II collagenase (C6685), LPS (L2880), cortisol (H0888), and Se (S5261) were all purchased from Sigma-Aldrich. Fetal bovine serum (10099141C) was purchased from Gibco. The CCK-8 assay kit (A311), cell cycle kit (C1052), and apoptosis analysis kit (C1062M) were all purchased from Beyotime. TRIzol (ET111), reverse transcription reagent kit (AT341), and quantitative PCR reagent kit (AQ601) were purchased from TransGen. β-actin (4970, 1:1000), p-PI3K (4228, 1:1000), PI3K (4292, 1:1000), p-AKT (4060, 1:2000), AKT (4691, 1:1000), c-Myc (5605, 1:1000), and Cyclin-D1 (2978, 1:1000) were purchased from Cell Signaling Technology. β-catenin (ab32572, 1:5000), p-GSK-3β (ab32391, 1:5000), and GSK-3β (ab75814, 1:10000) were obtained from Abcam. BAX (50599-2-Ig, 1:2000), Bcl-2 (26593-1-AP, 1:1000), and Lamin B1 (12987-1-AP, 1:5000) were purchased from Proteintech Biotechnology. HRP-conjugated goat anti-rabbit IgG (RS0002, 1:10000) was obtained from Immunoway. HRP-conjugated goat anti-mouse IgG (330, 1:10000) was obtained from MBL Beijing.

### 2.2. Isolation and Culture of Primary BESCs

The experimental procedures were reviewed and approved by the Animal Ethics Committee of Yangzhou University. In our previous studies, we have outlined the protocols for collecting bovine uterine samples [25]. Six healthy dairy cows were used for the isolation and culture of BESCs in this study. The healthy cow uteri in the pre-estrus stage were collected and placed in a cooler before being promptly transported to the laboratory. They were then disinfected with iodine and transferred to a biosafety cabinet for further manipulation. The uterine horns were surgically incised using sterile scissors to collect the endometrium, which was then repeatedly washed in PBS containing antibiotics. The collected endometrial samples were cut into approximately 1 mm^3^ tissue fragments and incubated with 0.4% collagenase at 37 °C for 50 min. The digested solution was centrifuged at 100× *g* for 5 min after filtering it through a 150 μm mesh sieve. Then, the cells were seeded into 25 cm^2^ culture flasks at a density of 1 × 10^6^ cells and incubated in a cell culture incubator at 37 °C and 5% CO_2_ in DMEM/F12 medium supplemented with 10% fetal bovine serum. To separate BESCs from BEECs, the cell suspension was removed after 18 h of culture, at which point BESCs adhered to the surface of cell culture flask while BEECs remained non-adherent [6]. The medium was refreshed every 48 h until BESCs reached 90% confluence, following which the cells were passaged.

### 2.3. Experimental Design

The study was divided into six groups: the control group, the LPS group, the LPS + COR group, and the LPS + COR + Se at concentrations of 1, 2, 4 μM. The BESCs were co-treated with LPS (10 μg/mL) and cortisol (30 ng/mL) for 24 h after pre-treatment with different concentrations of Se for 12 h. In the control group, an equal volume of DMEM/F12 medium was added, consistent with the treatment groups. The concentrations of LPS, Se, and cortisol have been verified in previous studies [24].

### 2.4. Cell Viability Assay

BESCs were seeded onto a 96-well culture plate at a density of 2 × 10^4^ cells per well and allowed to culture for a period of 24 h before being treated based on experimental design. Following treatment, 10 μL of the CCK-8 solution was added to each well, and the plates were then incubated for 2 h. The optical density at 450 nm was quantified with a microplate reader.

### 2.5. Western Blot Analysis

BESCs were inoculated into 6-well cell culture plates with 5 × 10^5^ cells per well. After treatment, total protein was extracted using RIPA lysis buffer, while nuclear protein was extracted using a nuclear protein extraction kit. The concentration of proteins was then measured with the BCA assay method. Protein samples (20–30 μg) were equally loaded onto a 10% polyacrylamide gel for electrophoretic separation, and subsequently moved onto a PVDF membrane. Following transfer, a 5% skim milk solution was applied to treat the membrane at room temperature for 1.5 h in order to minimize non-specific binding. Primary antibodies (β-actin, p-PI3K, PI3K, p-AKT, AKT, p-GSK-3β, GSK-3β, c-Myc, Cyclin-D1, β-catenin, Lamin B1, Bcl-2, and BAX) were applied and the membrane was incubated overnight at 4 °C, after which it was washed with TBST. Subsequently, the membrane was incubated with secondary antibodies, specifically HRP-conjugated goat anti-rabbit IgG or anti-mouse IgG, at room temperature for 1.5 h. This was followed by washing the membrane with TBST. The target proteins were visualized utilizing an Enhanced Chemiluminescence (ECL) reagent. Quantitative analysis of the relative band intensity of the target proteins was conducted with ImageJ software (ImageJ 1.53q, Bethesda, MD, USA).

### 2.6. Quantitative Real-Time PCR (qRT-PCR)

BESCs were inoculated into 6-well cell culture plates (5 × 10^5^ cells/well). Following treatment, the RNA was isolated with the TRIzol method and converted into complementary DNA (cDNA) through reverse transcription. The levels of growth factors gene expression in BESCs were quantified via qRT-PCR under specific thermal cycling conditions, starting with denaturation at 94 °C for 30 s, followed by denaturation at 94 °C for 5 s and annealing at 60 °C for 30 s. This cycle was repeated a total of 40 times. The specific primer sequences for β-actin, vascular endothelial growth factor (VEGF), connective tissue growth factor (CTGF), transforming growth factor beta1 (TGF-β1), and transforming growth factor beta 3 (TGF-β3) are provided in Table 1, with β-actin utilized as the internal control gene for normalization. The 2^−ΔΔCt^ method utilized for calculating the gene expression fold change in the target gene.

### 2.7. Cell Cycle Analysis

BESCs were seeded into 6-well cell culture plates at a density of 5 × 10^5^ cells per well. After treatment, BESCs were collected and rinsed in pre-chilled PBS, followed by fixation in 70% ethanol at 4 °C for 24 h. Subsequently, they underwent two additional washes with PBS. A 0.5 mL propidium iodide staining solution was added, followed by incubation at 37 °C for 30 min without exposure to light. Flow cytometry was employed to determine the cell cycle stage.

### 2.8. Apoptosis Analysis

BESCs were inoculated into 6-well cell culture plates with 5 × 10^5^ cells per well. After being treated, BESCs were washed with PBS, followed by the addition of 195 μL of Annexin-FITC binding buffer for resuspension. Next, the mixture was homogenized by carefully adding 5 μL of Annexin V-FITC and 10 μL of PI staining solution. The mixture was then incubated in the dark at room temperature for 10 min. Finally, the apoptotic BESCs were assessed utilizing flow cytometry.

### 2.9. Statistical Analysis

Each experiment was conducted in triplicate. The mean ± standard error of the mean (SEM) was utilized for presenting all data, which were analyzed with IBM SPSS Statistics 19 software (IBM, Armonk, NY, USA). Statistical significance was evaluated through one-way ANOVA followed by the least significant difference tests to determine differences between groups. A *p* value below 0.05 was considered to indicate statistical significance.

## 3. Results

### 3.1. Se Promoted the Viability of BESCs

**Figure 1:**
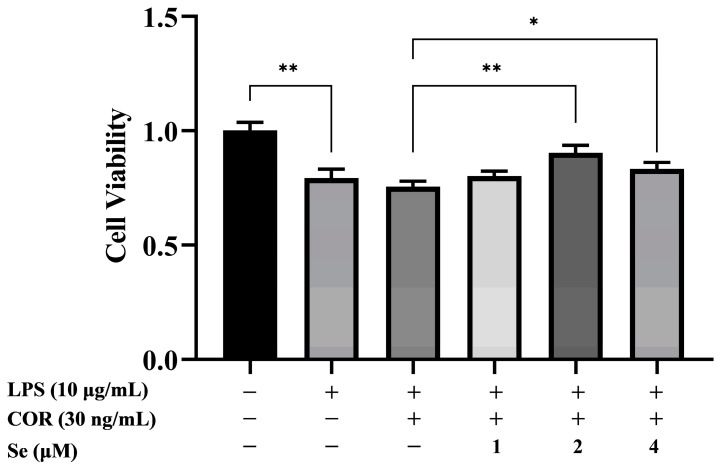
Effects of Se on viability of BESCs were confirmed by CCK-8 assay. * *p* < 0.05, ** *p* < 0.01.

The impacts of Se on cell viability in LPS-stimulated BESCs under high cortisol levels were detected using CCK-8. As illustrated by Figure 1, BESCs viability decreased due to LPS stimulation. BESCs viability increased with 2 μM and 4 μM Se treatment compared to the LPS+COR group. The data indicated that Se enhanced the viability of LPS-stimulated BESCs under high cortisol levels.

### 3.2. Se Increased the mRNA Levels of Growth Factors in BESCs

**Figure 2:**
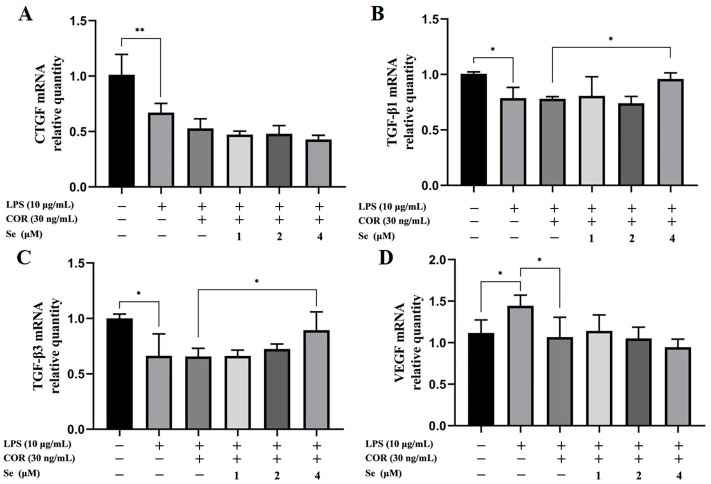
Impacts of Se on the mRNA level of CTGF (**A**), TGF-β1 (**B**), TGF-β3 (**C**), and VEGF (**D**) in BESCs was assessed by qRT-PCR analysis of extracted RNA. * *p* < 0.05, ** *p* < 0.01.

To confirm the effects of Se on BESCs proliferation, the mRNA levels of VEGF, CTGF, TGF-β1, and TGF-β3 were examined by qRT-PCR (Figure 2). The mRNA levels of CTGF, TGF-β1, and TGF-β3 decreased due to LPS stimulation, whereas VEGF expression increased. In comparison to the LPS group, the mRNA level of VEGF decreased in the LPS+COR group. However, 4 μM Se upregulated the mRNA levels of TGF-β1 and TGF-β3 compared to the LPS+COR group. Therefore, Se demonstrated a promoting effect on the gene levels of TGF-β1 and TGF-β3 in LPS-stimulated BESCs under high cortisol levels.

### 3.3. Se-Attenuated Apoptosis of BESCs

**Figure 3:**
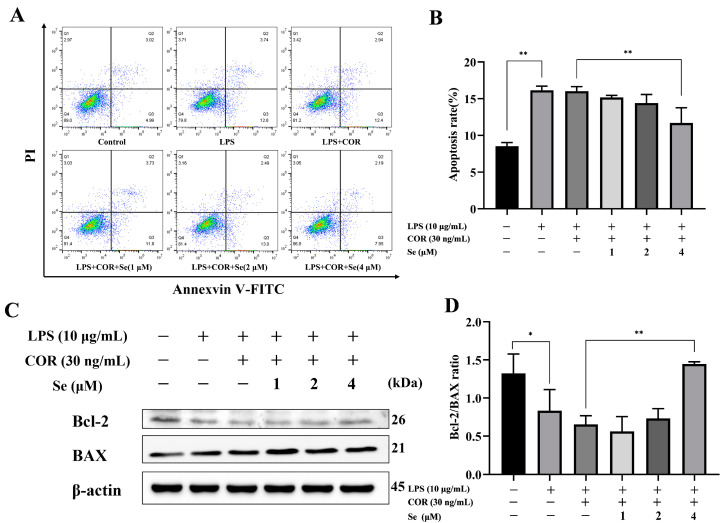
Effects of Se on cell apoptosis of BESCs. (**A**) The detection of cell apoptosis was performed using flow cytometry. (**B**) Quantitative analysis of the cell apoptosis rate. (**C**) The expression of Bcl-2 and BAX proteins was determined using Western blot analysis. (**D**) Quantitative analysis of the ratio of Bcl-2/BAX. * *p* < 0.05, ** *p* < 0.01.

The effects of Se on apoptosis in LPS-stimulated BESCs under high cortisol levels were detected using Annexin V/PI and Western blot analysis. Compared to the control group, the percentage of apoptotic cells increased and the ratio of Bcl-2 to BAX reduced due to LPS stimulation (Figure 3). A total of 4 μM Se reduced apoptotic cell count in comparison to the LPS + COR group. Additionally, treatment with 4 μM Se improved the ratio of Bcl-2 to BAX. The findings indicated that Se protected BESCs against apoptosis induced by LPS and cortisol.

### 3.4. Se Promoted the Cell Cycle of BESCs

**Figure 4:**
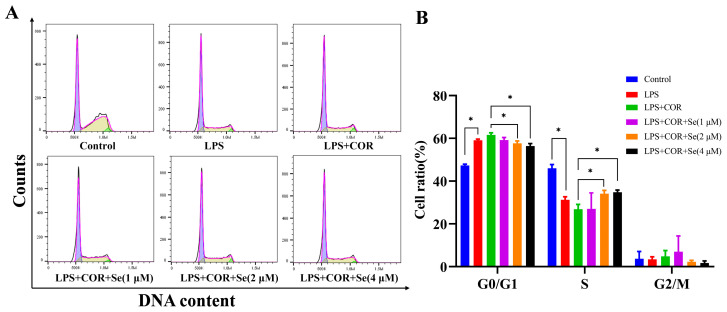
The role of Se in modulating cell cycle distribution in BESCs. (**A**) Flow cytometry was utilized to analyze the cell cycle progression. (**B**) Quantification of the cell cycle distribution. * *p* < 0.05.

To assess the potential impact of Se on BESCs proliferation, flow cytometry was utilized to analyze the cell cycle distribution. The results presented in Figure 4 demonstrated that LPS treatment led to an increase in the proportion of cells in the G0/G1 phase and a decrease in the proportion of cells in the S phase. Conversely, the proportion of cells in the G0/G1 phase was downregulated while the number of cells in the S phase was upregulated following treatment with 2 μM and 4 μM Se in comparison to the LPS + COR group. The above data suggested that Se may enhance cell proliferation via accelerating the progression into the S phase from the G0/G1 phase in LPS-stimulated BESCs under high cortisol levels.

### 3.5. Se Activated PI3K/AKT/GSK-3β Signaling Pathway of BESCs

**Figure 5:**
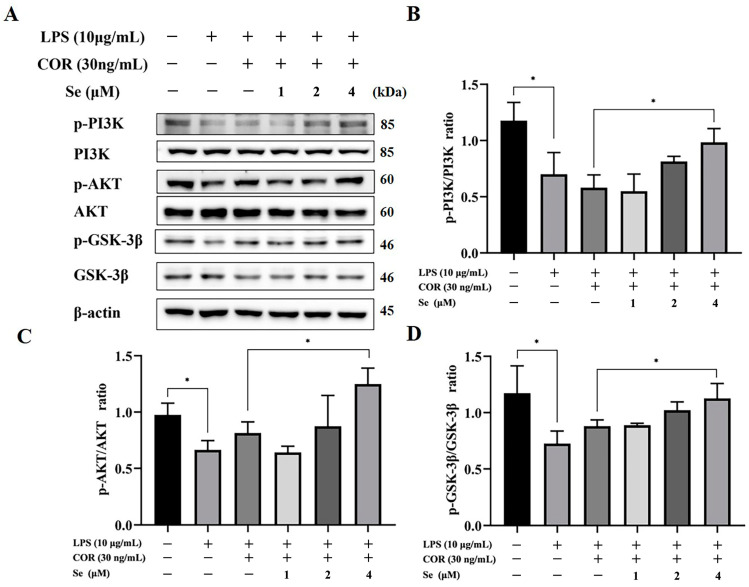
The role of Se in modulating the PI3K/AKT/GSK-3β signaling pathway of BESCs. (**A**) The phosphorylation levels of PI3K, AKT, and GSK-3β was evaluated using Western blot analysis. (**B**–**D**) Quantitative analysis of PI3K, AKT, and GSK-3β phosphorylation. * *p* < 0.05.

To explore whether Se can activate the PI3K/AKT/GSK-3β signaling pathway, the levels of PI3K, p-PI3K, AKT, p-AKT, GSK-3β, and p-GSK-3β were assessed via Western blot. As illustrated in Figure 5, phosphorylation of PI3K, AKT, and GSK-3β decreased due to LPS stimulation. However, elevated levels of phosphorylation of PI3K, AKT, and GSK-3β were identified following treatment with 4 μM Se. The above data demonstrated that Se can recover the activity of PI3K/AKT/GSK-3β signaling pathway, which had been suppressed due to LPS and cortisol.

### 3.6. Se Triggered the Activity of the Wnt/β-Catenin Signaling Pathway in BESCs

**Figure 6:**
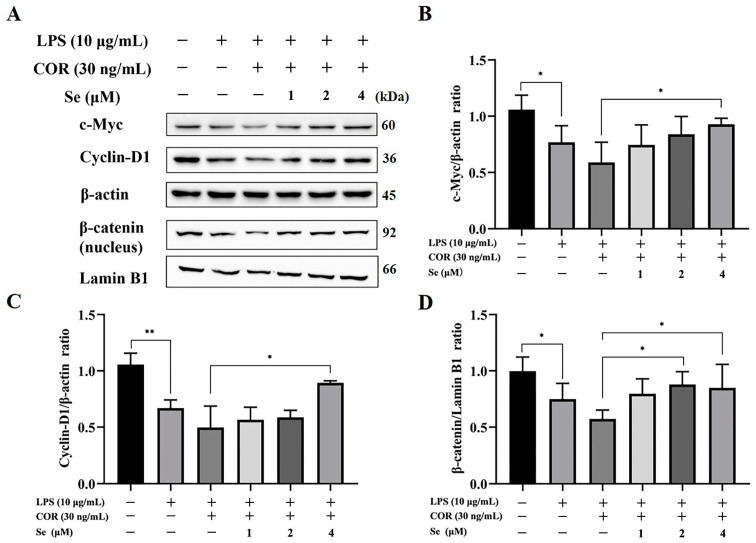
Effects of Se on the Wnt/β-catenin signaling pathway of BESCs. (**A**) The protein expression level of c-Myc, Cyclin-D1, and β-catenin was evaluated using Western blot analysis. (**B**–**D**) Quantitative analysis of c-Myc, Cyclin-D1, and β-catenin level. * *p* < 0.05, ** *p* < 0.01.

Western blot analysis was conducted to evaluate the protein expression level of β-catenin, c-Myc, and Cyclin-D1, in order to determine the regulatory impacts of Se on the Wnt/β-catenin signaling pathway. As illustrated in Figure 6, LPS administration suppressed the protein expression level of β-catenin, c-Myc, and Cyclin-D1. Conversely, treatment with 4 μM Se reversed the protein expression levels of β-catenin, c-Myc, and Cyclin-D1 that were inhibited by LPS and high levels of cortisol. These data suggested that the Wnt/β-catenin signaling pathway, which was suppressed by LPS and cortisol, was activated by Se.

## 4. Discussion

Endometritis in cows is one of the common diseases that can diminish their reproductive ability and milk yield [26]. The repair of the postpartum endometrium is vital for restoring the normal physiological function of the bovine uterus [27]. Wound repair involves a complex process in which cell proliferation, migration, apoptosis, and differentiation play pivotal regulatory roles [28,29]. The present results demonstrated that 10 μg/mL LPS inhibited the proliferation of BESCs while promoting cell apoptosis. Consequently, this concentration of LPS was used for further study. Se is a crucial microelement for livestock and plays a significant function in their normal growth and development. Previous studies have proved that Se could promote the growth of bovine endometrial epithelial cells [30]. That finding was consistent with our findings, which showed that LPS and cortisol caused the inhibition of BESCs proliferation, while Se stimulated cell proliferation. Wang et al. also suggested that an appropriate amount of Se could maintain neurocyte viability and inhibit cell apoptosis [31]. In line with this study, we found that 4 μM Se reduced the number of apoptotic BESCs induced by LPS and cortisol. Apoptosis, also recognized as programmed cell death, is an essential physiological process necessary for normal development and the maintenance of homeostasis [32]. Components of the Bcl family, like BAX and Bcl-2, recognized as pro- or anti-apoptotic proteins, respectively, are located on the mitochondrial membrane and serve as pivotal mediators of the intrinsic apoptotic pathway [33]. BAX facilitates cell death by inducing permeability in the outer mitochondrial membrane in response to various cellular stresses, whereas Bcl-2 inhibits apoptosis by blocking BAX activity [34]. Western blot analysis indicated a notable enhancement in the Bcl-2-to-BAX ratio with Se treatment compared to treatment with LPS and cortisol, further confirming the anti-apoptotic effect of Se. Thus, Se could counteract the alterations of proliferation and apoptosis damaged by LPS and cortisol.

The process of tissue repair is dynamic and sequential, controlled by a myriad of potent biomolecules known as growth factors. Connective tissue growth factor (CTGF) has been identified as a fibrosis marker in endometrial diseases [35]. In the condition of wound repair, CTGF overexpression enhances wound healing, boosts the proliferation of connective tissue cells, and facilitates cell attachment. Vascular endothelial growth factor (VEGF) stimulates cell growth and inhibits apoptosis in endothelial cells, enhances vascular permeability, and stimulates angiogenesis [36]. Transforming growth factor-β (TGF-β) consists of multifunctional growth factors that regulate invasion, migration, epithelial–mesenchymal transition, apoptosis, and wound healing [37]. The crucial role of TGF-β1 in endometrial growth have been demonstrated by many studies [38,39]. The study exhibited that Se could increase the mRNA level of TGF-β1 and TGF-β3, which were inhibited by LPS and cortisol. Similarly to this result, Nafiu et al. verified that Se contributed to wound healing by the transient increase in the expression of TGF-β1 combined with unripe Carica papaya pulp extracts in rats [40]. Conversely, VEGF mRNA expression was higher with LPS treatment; however, its overexpression was reversed by cortisol treatment. This may be attributed to the significant release of inflammatory factors induced by inflammatory stimulation. There is ample strong evidence indicating that LPS-induced inflammation leads to VEGF overexpression [41,42]. Thus, our results revealed that Se may accelerate BESCs growth via restoring expression of TGF-β1 and TGF-β3.

As is commonly recognized, cell proliferation and apoptosis are tightly regulated by the cell cycle. The arrest of cell cycle progression in the G0/G1 phase prevented the cells from advancing into the S phase. Liu et al. suggested that the transition from the G1 to the S phase was delayed by LPS in bovine mammary epithelial cells, whereas selenomethionine restored the alterations [43]. Consistent with this finding, our data indicated a decrease in the proportion of BESCs in the G0/G1 phase and an increase in the proportion of BESCs in the S phase using Se treatment, suggesting that Se promotes cell cycle progression hindered by LPS and cortisol.

The PI3K/AKT/GSK-3β and Wnt/β-catenin signaling pathways were demonstrated to be involved in signal transduction, activating a cascade of growth signaling while suppressing apoptotic pathways, thereby facilitating cell proliferation and survival [44,45]. Se additives downregulated the inflammatory reaction and increased cell proliferation via regulating the PI3K/AKT/GSK-3β signaling pathway in LPS-induced bovine endometrial epithelial cells, as well as downregulating cell apoptosis in Pb-induced chicken splenic lymphocytes [24,46]. These results were consistent with our results, as Se upregulated the level of p-PI3K, p-AKT, and p-GSK-3β in BESCs. Previous studies have proved that activating the PI3K/AKT signaling pathway enhanced the proliferation of endometrial cells [47]. The phosphorylation of PI3K and AKT is a process involving key molecules in the PI3K/AKT signaling pathway. Upon activation, p-AKT phosphorylates the downstream protein GSK-3β; additionally, p-GSK-3β serves as a canonical negative modulator in the Wnt signaling pathway by suppressing the activation of β-catenin and impeding its nuclear translocation, ultimately hindering the activation of the Wnt/β-catenin signaling pathway [48]. In the Wnt/β-catenin signaling pathway, β-catenin, c-Myc, and Cyclin-D1 are pivotal components, with c-Myc and Cyclin-D1 serving as target genes directly involved in cell proliferation and differentiation [49]. Research findings have established the essential roles of Cyclin-D1 in the G1/S phase transition and c-Myc in the G2/M phase transition [50,51]. In the current study, Se was found to activate the Wnt/β-catenin signaling pathway, leading to elevated c-Myc and Cyclin-D1 expression. These results were also confirmed by the reversal of cell cycle arrest induced by LPS and cortisol through the treatment of Se in BESCs. Taken together, Se’s influence on cell proliferation and apoptosis may be connected to the PI3K/AKT/GSK-3β and Wnt/β-catenin signaling pathways.

## 5. Conclusions

In conclusion, our results demonstrated that Se could enhance cell proliferation and suppress apoptosis caused by LPS and cortisol. Furthermore, we revealed the protective effect of Se on BESCs may be achieved through the activation of the PI3K/AKT/GSK-3β and Wnt/β-catenin signaling pathways, which contributed to the rapid repair of the postpartum endometrium.

## Figures and Tables

**Table 1:** The primer sequences utilized in qRT-PCR.

Names	Primers (5′→3′)	Product Sizes (bp)	Accession Number
β-actin-F	CATCACCATCGGCAATGAGC	156	NM_173979.3
β-actin-R	AGCACCGTGTTGGCGTAGAG		
VEGF-F	CCTGATGCGGTGCGGGGGCT	372	NM_001316992.1
VEGF-R	TGGTGGTGGCGGCGGCTATG		
CTGF-F	AGCTGACCTGGAGGAGAACA	139	NM_174030.2
CTGF-R	GTCTGTGCACACTCCGCAGA		
TGF-β1-F	CGAGCCCTGGACACCAACTA	137	NM_001166068.1
TGF-β1-R	AGGCAGAAATTGGCGTGGTA		
TGF-β3-F	CTGTGCGTGAATGGCTCTTG	153	XM_005212206.5
TGF-β3-R	CATCATCGCTGTCCACACCT		

## Data Availability

The data presented in this study are available in the article.
